# Clinical Assessment of Fluid Status in Adults With Acute Kidney Injury: A Scoping Review

**DOI:** 10.1111/jorc.70014

**Published:** 2025-04-05

**Authors:** Karen Nagalingam, Lisa Whiting, Ken Farrington, Janet Migliozzi, Natalie Pattison

**Affiliations:** ^1^ University of Hertfordshire Hatfield UK; ^2^ Lister Hospital, East and North Hertfordshire NHS Trust Stevenage UK

**Keywords:** acute kidney injury, nursing assessment, signs and symptoms, symptom assessment

## Abstract

**Background:**

Acute kidney injury refers to sudden, potentially reversible, reduction in kidney function. Hypovolaemia is commonly the major risk factor. When acute kidney injury is established, fluid can accumulate leading to fluid overload. Undertaking a rigorous fluid assessment is vital in the management of a patient in hospital with acute kidney injury, as insufficient or excessive fluid can lead to increased morbidity and mortality.

**Objectives:**

The aim of this scoping review is to identify which clinical assessments are useful when undertaking fluid assessment in a patient with acute kidney injury, and to identify signs and symptoms of fluid overload or dehydration in patients in hospital with acute kidney injury.

**Design:**

The JBI methodology for scoping reviews was followed and reported using the PRISMA‐ScR checklist. PubMed, CINAHL Plus and SCOPUS were searched for research papers relating to the signs and symptoms or fluid assessments in patients with acute kidney injury.

**Results:**

Fifteen research papers were identified with four key areas being: Fluid balance/urine output and weight; early warning scores; clinical signs and symptoms; holistic assessment. The primary studies included in this scoping review have shown that hypovolaemia may be indicated by low blood pressure, orthostatic hypotension, low Mean Arterial Pressure, elevated heart rate, prolonged capillary refill time on the sternum (> 4.5 s) and subjectively reported cold peripheries. With clinical symptoms including dry mouth, increased thirst and dry skin. Accurate documentation of urine output and fluid balance is crucial in determining fluid status.

**Conclusion:**

The assessment of fluid should be holistic and include history taking, diagnosis, blood tests and associated clinical signs and symptoms.

## Summary

- Patients in hospital with acute kidney injury (AKI) should have accurate documentation of urine output and fluid balance.
- A patient who has prolonged hypotension or has orthostatic hypotension is at risk of AKI developing and/or progressing.
- Useful indicators of dehydration in a patient with AKI include low blood pressure, orthostatic hypotension, increased heart rate, prolonged capillary refill time subjectively cold extremities, dry mouth, increased thirst and dry skin.

## 1 Introduction

Acute kidney injury (AKI) is present in around 8%–16% of hospital admissions in the UK (Sawhney et al. 2017), and approximately 1 in 5 admissions worldwide (Susantitaphong et al. 2013). AKI is usually secondary to other health problems and is frequently multifactorial (Chawla et al. 2017), with causes categorised into pre‐renal (reduction in volume or blood flow to the kidney), intrarenal (injury to the nephron) or post‐renal (urinary flow blockage) (Yang et al. 2014).

Variations in cause may be attributable to environmental and socioeconomic reasons (Rewa and Bagshaw 2014) but pre‐renal factors remain the most common cause of AKI worldwide (Mehta et al. 2016). A reduction in blood flow to the kidney can be attributed to (a) hypovolaemia (NICE 2019) which is when there is a reduction in effective circulating volume, (b) dehydration which is when there is a reduction in total body water (Kitiwan et al. 2021) or (c) relative hypovolaemia (Patil and Salunke 2020) where fluid is in the tissues and not in the vascular system (sepsis and heart failure). Whilst these terms all have different meanings, they all result in a reduction in renal blood flow. Therefore, early fluid resuscitation is deemed crucial in these individuals to prevent further deterioration and to minimise the risk of intrinsic kidney damage developing (Molitoris 2022). Excess fluid in patients with AKI is also a causal factor in increased risk of death (Zhang et al. 2015; Neyra et al. 2016).

Basic fluid management is a fundamental requirement for managing patients who are experiencing clinical deterioration (Bednarczyk et al. 2017; Rochwerg et al. 2016; Resuscitation Council UK 2020). The identified gold standard fluid assessment of an acutely ill adult is defined by the National Institute for Health and Care Excellence (NICE 2013) and includes measurement of physiological parameters, physical examination and history taking. There is also further guidance for physicians on undertaking fluid assessment in patients in hospital with AKI found in the *“Acute care toolkit 12: Acute kidney injury and intravenous fluid therapy”* (Royal College Of Physicians 2015). This cites blood pressure, pulse, respiratory rate, capillary refill time (CRT), jugular venous pressure (JVP), oedema, passive leg raises, fluid balance chart review, and weight as useful in the assessment of patients with AKI. However, not all of these assessments are feasible to undertake in this patient group because of issues such as frailty. In addition, common comorbidities such as obesity, heart failure and hypoalbuminaemia can create difficulty in interpreting the clinical picture of fluid status (Frank Peacock and Soto 2010). Rapid accurate assessment of fluid status was outlined as critical for effective management of AKI by the 2009 National Confidential Enquiry into Patient Outcomes and Deaths (NCEPOD) report (NCEPOD 2009). Elements that could be utilised in a fluid assessment are outlined by NICE (2013), although clinical assessment of fluid is variable, with a range of terms used (Bond and Fletcher 2012). Subjective physical examination of volume status is difficult, with Steéphan et al. (2001) stating that single clinical signs where not useful in identifying hypovolaemia in critically ill patients, and some clinical signs having little value in identifying hypovolaemia (Pacagnella et al. 2013). There is no consensus for determining volume status in patients with AKI (Bond and Fletcher 2012). The consequences of inaccurate assessment include too little or too much fluid being administered, with concomitant risks of increased morbidity and mortality (Connell and Laing 2015).

To identify if there were any current systematic or scoping reviews focusing on fluid assessment in patients with AKI, a preliminary search of Scopus, the Cochrane Database and JBI Evidence Synthesis was undertaken with no positive outcome and very little other specific literature. Therefore, the objective was to assess the literature relating to fluid assessment as well as the signs and symptoms exhibited by patients with AKI in hospital. Whilst systematic reviews may be valuable to answer specific questions and provide reliable findings to draw conclusions from, scoping reviews provide an overview of the evidence (Munn et al. 2018). With limited specific literature, a scoping review was identified as being the most appropriate method.

## 2 Methods

### 2.1 Study Design

A scoping review was undertaken to present an overview of a topic from a broad body of literature (Pollock et al. 2021). This approach is ideal for informing practice and for identifying knowledge gaps (Munn et al. 2018). Arksey and O'Malley (2005) provide a framework for scoping reviews comprising of five steps: (1) Identifying the research question, (2) identifying relevant studies, (3) eligibility, (4) charting the data and (5) reporting the results.

### 2.2 Identification of the Research Question

The scoping review was designed to answer the question: *What signs and symptoms of fluid status (overload/dehydration) are exhibited in adult patients with AKI and what assessments provide an accurate assessment of fluid status?*

### 2.3 Identification of Relevant Studies

To structure the research question and to develop the search terms, “PICO” was used (Stern et al. 2014) (see Table 1). All relevant studies were identified using an electronic literature search. An initial search was conducted in March 2023 and then again April 2024. The databases used were PubMed, CINAHL Plus and SCOPUS. The search was undertaken using three concepts: 1. Acute kidney injury, 2. Fluid assessment and 3. Signs and symptoms. The search used combined concepts as well as the Boolean operator “AND”; there were many research papers found within PubMed; however, no literature was found in CINAHL Plus or SCOPUS and, therefore, Concepts 2 “AND” 3 and Concepts 1 “AND” 2 were included in the search. In addition to this, the Boolean operator “OR” was used to include the key words: Weight, hypotension, capillary refill time (CRT), early warning scores (EWS), respiratory rate, blood pressure (BP), dehydration, oedema, jugular venous pressure (JVP) and euvolaemia (see Table 1). A clinical librarian reviewed the search strategy.

**Table 1:** Search terms for the scoping review.

	Inclusion	Exclusion	Concept	MeSH	Other search terms identified
Population	Adult patients with AKI	≤ 18 years, no confirmation of AKI, pregnancy, end of life, chronic kidney disease,	Acute kidney injury	(“acute kidney injury/complications” OR “acute kidney injury/nursing” OR “acute kidney injury/physiopathology” OR “acute kidney injury/urine”)	“Acute kidney injury” OR “acute renal failure” OR AKI
Intervention	Fluid assessment, capillary refill time, pulse rate, respiratory rate, oedema, blood pressure, jugular venous pressure, weight, National early warning score (NEWS)	No fluid assessment undertaken	Fluid assessment	“Symptom assessment” AND “nursing assessment”	“Symptom assessment” AND “nursing assessment” OR hypovol* OR dehydrat* OR euvol* OR hypervol* OR “fluid overload*” OR NEWS* OR “capillary refill time” OR “jugular venous pressure” OR JVP OR weight OR hypotensi* OR “respiratory rate” OR Edema* OR oedema*
Comparison	Other approaches used to assess fluid status, signs and symptoms		Signs and symptoms	“Signs AND symptoms”[Mesh]	“Signs and symptoms” OR hypotensi* OR “respiratory rate” OR hypertensi* OR “weight” OR edema* OR oedema*
Outcomes	Patient is fluid overloaded, euvolaemic, or hypovolaemic				

Tables of contents of renal specific journals were also searched, and snowballing was used to retrieve other papers from the references of articles. Google Scholar was also used to locate additional papers.

The reporting of the scoping review follows Preferred Reporting Items for Systematic Review and Meta‐Analyses Extension for Scoping Reviews (PRISMA‐ScR) guidelines (Tricco et al. 2018) and the article selection process is shown below.

### 2.4 Inclusion/Exclusion Criteria

Inclusion criteria for the research articles included: adults over the age of 18 years; being written in English with a retrievable full text. Literature focussing on pregnancy or case reports were excluded. An initial scan of the title identified any duplicates and articles not fitting the inclusion criteria (including secondary literature)—these were removed. No date limitations were set.

### 2.5 Study Selection and Screening, Data Charting

Papers that were included were original research was in the English language, but with any setting and study design. Although the focus was AKI, if a study included this as a secondary outcome it was deemed sufficient to incorporate within the scoping review. Full‐text screenings were undertaken on papers that were thought to meet the eligibility criteria. A data charting form was developed from the JBI Methodology for scoping reviews guidance (Peters et al. 2015b), which included: author, year of publication and location, aims of the study, population and size of sample, methodology, interventions and follow up, outcomes and key findings (Peters et al. 2015a). The data were independently extracted by two reviewers, who then discussed which papers should be included in the review (KN and JM).

**Figure 1:**
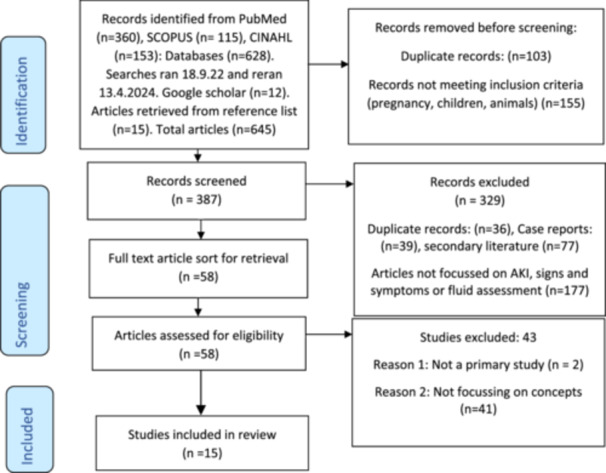
PRISMA 2020 flow diagram: For enhanced fluid assessment study. *From:* Page MJ, McKenzie JE, Bossuyt PM, Boutron I, Hoffmann TC, Mulrow CD, et al. The PRISMA 2020 statement: an updated guideline for reporting systematic reviews. BMJ 2021;372:n71. doi: 10.1136/bmj.n71. For more information, visit: http://www.prisma-statement.org/. See Supporting Information.

The article selection process is shown in Figure 1.

### 2.6 Analysis

The papers were systematically and comprehensively examined (Ryan and Hill 2016), organised into themes and the results tabulated (Table 2). The findings are described below using a narrative synthesis; study characteristics are summarised and include type of study, country, population and number of participants as well as the aim.

**Table 2:** Research articles from the scoping review.

Theme	Author/year	Aim	Methodology	Numbers of participants	Key findings
Fluid balance (FB)/urine output and weight	Baggot et al. (2023)	To identify if oliguria is a better measurement for identifying AKI stage 1 than creatinine measurement, using electronic fluid balance (FB) charts	A single centre prospective cohort study	2149 patients on a hospital ward setting in England	Oliguria represented an early sign of kidney disfunction. Although some of the FB data was not accurate due to missing data
	Wang et al. (2022)	To identify different trajectories of fluid balance on outcome of patients in Intensive care unit (ICU) with AKI	Multicentre, prospective observational study	1529 Critically ill patients with AKI, in 30 ICUs at 28 tertiary hospitals in Beijing in China	Decreasing fluid balance were associated with reduction in 28‐day mortality
	Chow et al. (2020)	To assess weight as a method of identifying fluid overload	Small observational pilot study	58 patients on a general ward in Singapore	Percentage of fluid overload by weight increase (PFO_w_) of ≥ 1% was consistently and strongly associated with Stage 2 or 3 AKI.
	Jin et al. (2017)	Intensity of urine output monitoring on outcomes in ICU patients with AKI	Multicentre, retrospective cohort review	15,724 patients in ICU in America	Intensive monitoring of urine improved detection of AKI and reduced 30‐day mortality
	Lee et al. (2015)	To examine the association between fluid balance and survival	Longitudinal single centre study examining fluid balance at discharge.	15,395 patients discharged from ICU in America	Positive fluid balance was a statistically significant predictor of 90‐day mortality
	Teixeira et al. (2013)	To evaluate the impact of fluid balance and urine volume on outcome of ICU patients with AKI	Multicentre, prospective cohort study	132 patients in 10 Italian ICUs	Low urine volume and higher mean fluid balance were associated with increased risk of death
	Prowle et al. (2011)	To evaluate the relationship between AKI and oliguria	Multicentre, prospective cohort study	239 patients. Six countries (Australia, Canada, Japan, USA, Germany, Italy) in seven ICUs. 32 patients had AKI on admission. 23 developed AKI during admission	Oliguria was statistically significantly associated with the development of AKI
Early warning scores	Faisal et al. (2018)	To predict hospital acquired AKI (H‐AKI) by monitoring vital signs as defined by NEWS	Single centre retrospective cohort study	33,608 emergency admissions in England	NEWS is a poor predictor of AKI; elevated serum potassium, and deranged levels of bicarbonate were more useful
	Potter et al. (2017)	To examine the role of NEWS and other factors in identifying critically ill AKI patients with negative outcomes	Single centre retrospective cohort study.	64 patients seen by the critical care outreach team in England	In patients with AKI, NEWS has little role in the escalation of the patient.
Clinical signs	Wiersema et al. (2020)	To establish the value of clinical examination of patients in ICU with AKI	Multicentre prospective observational cohort study	1003 critical care patients in the Netherlands	CRT, cold peripheries, heart rate were able to predict AKI with moderate accuracy
	Panuccio et al. (2020)	To assess the duration of hypotension and progression of AKI	Small pilot study	39 patients with AKI in Italy	Patients with orthostatic hypotension and supine hypotension also had symptoms of dehydration
	Mehta et al. (2016)	To assess recognition, management and outcomes of AKI in different countries	Multinational cross‐sectional study	72 countries and 289 centres with 4018 patients	Hypotension (40%) and dehydration (38%) were the most common causes of AKI with rates varying depending upon classification of higher/higher middle/lower middle or lower‐income countries
	Izawa et al. (2016)		Single centre retrospective observational study	538 patients in ICU in Japan	The risk of AKI decreased as MAP increased (65, 70 and 75 mmHG)
	Poukkanen et al. (2013)	To examine whether higher MAP during the first 24 h is associated with lower risk of AKI progression	Multicentre prospective observational study	423 patients in the ICU with severe sepsis in Finland	A MAP of less than 73 mmHg in patients with severe sepsis, was associated with progression of AKI.
Clinical assessment	Lloyd et al. (2019)	To describe an approach to assessing patients requiring fluid resuscitation	Qualitative study, semi‐structured interviews.	18 clinicians of varying experience in England	Different types of assessment were undertaken depending upon the type of setting

### 2.7 Findings

A total of 645 articles were identified. Titles and abstracts were scanned (KN) and 258 were initially removed as they did not fit the search criteria. The remaining 387 papers were then screened for duplicates, secondary literature sources were excluded as well as case reports, articles that focussed on pregnancy or children and any articles which were not focussed on AKI and signs and symptoms or fluid assessment (KN), leaving 58 articles. The abstracts were reviewed and 24 were identified as meeting initial eligibility criteria; these were charted using JBI methodology for scoping review guidance and were independently reviewed by two reviewers (KN; JM); with agreement on 15 papers to be included, six to be excluded, and three papers discussed at length with advice sought (NP, KF and LW) which led to these three being excluded. This left 15 key papers (Figure 1) which included studies published between 2011 and 2023 with five studies from the UK, and the remaining 10 from other countries worldwide. Seven of the studies were multicentre, five were single centre, two were pilot studies; 14 were quantitative (observational, no interventional) and one study was qualitative (semi‐structured interviews) (refer to Table 2).

### 2.8 Fluid Balance/Urine Output and Weight

Six articles delineated fluid balance and urine output in patients with AKI; of these, only one was conducted outside of a critical care unit (Baggot et al. 2023). Fluid balance was found to be important in outcomes for patients with AKI, with an increasing positive fluid balance and risk of death being reported in three studies (Wang et al. 2022; Teixeira et al. 2013; Lee et al. 2015). Two papers found that intensive monitoring of urine output improved detection of AKI (Prowle et al. 2011; Jin et al. 2017) as well as reducing 30‐day mortality and fluid overload (Jin et al. 2017). Oliguria was identified more frequently and earlier in the course of AKI (Prowle et al. 2011; Baggot et al. 2023). With the identification of oliguria being statistically significantly associated with AKI diagnosed with a raised creatinine (Prowle et al. 2011). However, using urine output alone to diagnose AKI is thought to be oversensitive as it does not always lead to raised creatinine (Prowle et al. 2011; Baggot et al. 2023) and outside of the critical care setting, assessment of fluid balance was found to be inaccurate with over detection of oliguria (Baggot et al. 2023).

Intense monitoring of urine (hourly recordings with no gaps greater than 3 h) may enable better fluid management in patients (Jin et al. 2017), as fluid can be tailored to the urine output and clinical need. A balanced fluid status at discharge from ICU was found to be associated with improved survival; a negative fluid balance improved survival in people with congestive heart failure who also had AKI (Lee et al. 2015).

One study found that ≥ 1% increase in weight may be predictive of progression of AKI to Stage 2 or 3 (Chow et al. 2020).

### 2.9 Early Warning Scores and AKI Alerts

Two papers examined National Early Warning Scores (NEWS) and their role in identifying AKI (Faisal et al. 2018; Potter et al. 2017); both found no evidence that NEWS was useful in identifying AKI. An episode of AKI in the previous 12 months and current AKI stage, may be superior to NEWS in identifying deterioration (Potter et al. 2017). Potter et al. (2017) found that 45% of the patients reviewed, who went on to develop Stage 3 AKI, were not escalated due to an elevated NEWS score and concluded that NEWS has little role in the escalation of the patient. Faisal et al. (2018) supported this conclusion and stated that deranged serum potassium and bicarbonate were more useful. Potter et al. (2017) found that patients may have normal creatinine for several days even with deranged potassium and bicarbonate that is indicative of renal injury.

### 2.10 Clinical Signs and Symptoms

Five studies explored clinical examination with four of these considering hypotension and its association with AKI (Poukkanen et al. 2013; Mehta et al. 2016; Izawa et al. 2016; Panuccio et al. 2020). Hypotension and dehydration are cited as the most common causes of AKI worldwide (Mehta et al. 2016). However, rates vary depending on classification of higher/higher middle/lower middle or lower‐income countries, but there is confirmation that pre‐renal factors remain the most common causes of AKI worldwide (Abebe et al. 2021). However, it was found that there was no clear consistent definition of hypotension within the findings. It was defined as a systolic BP of < 110 mmHg in the supine position or a fall in systolic BP of > 10 mmHg when standing in ward based patients (Panuccio et al. 2020) and as < 90 mmHg in patients who were in critical care (Poukkanen et al. 2013). In the study by Panuccio et al. (2020), 15 patients were identified as hypovolaemic with 7 patients having a postural drop of 22 mmHg (11–54 mmHg) and the remaining 8 had hypotension in the supine position (systolic BP < 110 mmHg) as well as other symptoms of dehydration. These included: dry mouth, increased thirst, dizziness and dry skin. Although this was a small‐scale study, it was the only one that identified a link between low blood pressure (supine position) and symptoms of dehydration in patients with AKI. However, this was the only paper that was not critical care based (Panuccio et al. 2020) and this may account for the higher BP reading for defining hypotension.

There were three studies that described a link between AKI and mean arterial pressure (MAP) (Poukkanen et al. 2013; Izawa et al. 2016; Redfors et al. 2011). Poukkanen et al. (2013) found that patients who had a hypotensive episode (defined as a MAP of under 73 mmHg over a time‐adjusted period) were more likely to have progression of their AKI. Izawa et al. (2016) also reported a similar finding in that the risk of AKI decreased as the MAP increased (65, 70 and 75 mmHg) although prolonged hypotension (defined as between 3 and 6 h) was significantly associated with AKI progression. Redfors et al. (2011) found that improving MAP from 60 to 75 mmHg led to an increase in glomerular filtration rate.

One study examined the predictive value of clinical examination for AKI in patients admitted to critical care (Wiersema et al. 2020). It was reported that increased heart rate (OR: 1.12 per 10 beats per minute increase, 98.5% CI: 1.04–1.22), had a linear relationship with AKI. CRT was measured on the finger, sternum and knee with pressure applied for 15 s (Lima et al. 2009). Prolonged CRT was defined as > 4.5 s (Lima et al. 2009; Wiersema et al. 2020) with prolonged CRT on the sternum (OR: 1.89, 98.5% CI: 1.01–3.55) being the most predictive. Finally, subjectively cold extremities (OR: 1.52, 98.5% CI: 1.07–2.16) was identified as a more useful indicator of AKI than measured temperature difference that was taken centrally, via the bladder, and peripherally on the foot (Wiersema et al. 2020).

### 2.11 Clinical Fluid Assessment

The one qualitative study examined fluid assessment practices; Lloyd et al. (2019) found that clinicians would alter their fluid assessment depending on how sick a patient was. An initial screening assessment, in the form of an end of the bed visual scan or focussed review of observations (possibly undertaken by a nurse or other colleague) would dictate how the fluid assessment would progress; this then led to either an emergency or screening assessment as well as a formal one. The emergency fluid assessment was closely associated with the ABCDE assessment, certain clinical signs (such as blood pressure or respiratory rate) and bedside investigations. The formal assessment was viewed by participants as the optimal approach for fluid assessment—this being a more in‐depth assessment of the patient that included patient history, examination, observations and patient blood investigations. Participants stated that blood pressure was important for the assessment of hypovolaemia and 83% (*n* = 15) mentioned pulse rate (Lloyd et al. 2019). Clinical signs deemed to be useful in undertaking a fluid assessment included: peripheral and sacral oedema; skin turgor; CRT; swollen abdomen; lying and standing blood pressure; JVP; pulse (volume, character and rate); temperature; sweating; consciousness; mucous membranes; the presence of sunken eyes; dry axilla and auscultation of the lungs and heart.

## 3 Discussion

This scoping review describes several assessments that are useful when undertaking a fluid assessment in patients with AKI. The key review findings show that these assessments can be grouped under four key areas: holistic assessment; fluid balance/urine output and weight; early warning scores and clinical signs and symptoms. The review identified that oliguria and reduced fluid balance (Wang et al. 2022; Teixeira et al. 2013; Prowle et al. 2011; Baggot et al. 2023) as well as fluid overload identified by fluid balance (Lee et al. 2015) and weight (Chow et al. 2020) were indicators of AKI and lead to worse outcomes, whereas intensive monitoring of urine improved outcomes and detection of AKI (Jin et al. 2017). NEWS was a poor predictor of developing AKI (Faisal et al. 2018) and not useful in escalating patients with AKI (Potter et al. 2017). However, other signs like prolonged CRT, cold peripheries, increased heart rate (Wiersema et al. 2020) and deranged electrolytes were more useful (Faisal et al. 2018), alongside hypotension (including supine/orthostatic) (Panuccio et al. 2020), reduced MAP (Poukkanen et al. 2013; Izawa et al. 2016) and dehydration (Mehta et al. 2016). Other findings included using a holistic approach to assessment (Lloyd et al. 2019) and considering patient symptoms (Panuccio et al. 2020).

### 3.1 Indicators of Assessment

#### 3.1.1 Holistic Assessment

The formal fluid assessment was described by Lloyd et al. (2019) as the optimal approach and alongside the clinical signs, symptoms and assessments, should include past medical history, current clinical status and risk factors for AKI. This holistic approach to fluid assessment may enhance decision making around fluid status (NICE 2019).

#### 3.1.2 Fluid Balance, Urine Output and Weight

Oliguria was identified as an indicator of AKI (Prowle et al. 2011) and, therefore, urine should be measured in patients with AKI and included within a fluid balance chart; with the overall goal being the balancing of fluid status as this improves survival (Lee et al. 2015). Conversely, a positive fluid balance is associated with increased mortality (Neyra et al. 2016; Lee et al. 2015; Wang et al. 2015; Wang et al. 2022; Teixeira et al. 2013). Whereas intensive monitoring of urine output may be possible in critical care units, on general wards this is often inadequate with missing data (Chow et al. 2020; Leinum et al. 2023), meaning that conclusions about oliguria may be inaccurate (Baggot et al. 2023).

Weight based assessment may be useful to predict worsening AKI (Chow et al. 2020) but may also provide an alternative medium for assessing fluid status over a period of time. Vivanti et al. (2008) found that weight changes over a week and changes in percentage of total body weight were in a direction consistent with changes in hydration status. Therefore, the use of weight as a measure of fluid status (Royal College Of Physicians 2015) may be a useful element and more feasible than maintenance of an accurate fluid balance record on general wards. However, acute reductions in urine output cannot be detected with this method and it may not be possible to weigh all patients.

#### 3.1.3 Clinical Signs, Symptoms and Early Warning Scores

Clinical signs and symptoms which are valuable in assessing fluid in a patient with AKI, include: blood pressure, heart rate, MAP, CRT, dry mouth, increased thirst, dizziness and dry skin. Blood pressure alone may be a useful indicator of pre‐renal AKI and, therefore, of hypovolaemia; however, there is no clear definition of hypotension (Sharma et al. 2024) and a lack of international consensus on the definition and diagnosis of dehydration (Lacey et al. 2019). In ward based patients, hypotension was signified by a systolic BP of < 110 mmHg in the supine position or a lying and standing BP with a drop in systolic BP of > 10 mmHg when standing (Panuccio et al. 2020). Whereas in critical care setting, hypotension was defined as a systolic of < 90 mmHg and by using MAP (Poukkanen et al. 2013) as a surrogate for BP; the length of time that a patient was hypotensive was statistically significantly associated with progression of AKI (Izawa et al. 2016). Recommendations for minimum MAP has been cited as ≥ 65 mmHg (Daniels and Nutbeam 2019; Dellinger et al. 2013) which is in the guidance from the Surviving Sepsis campaign and from the Sepsis Trust (Dellinger et al. 2013). As MAP readings increase, risk of AKI reduces (Izawa et al. 2016; Poukkanen et al. 2013); therefore, it may be useful to aim for a MAP ≥ 73 mmHg as it may be beneficial when considering fluid resuscitation in patients at risk of, or with, pre‐renal AKI.

Clinical signs that are cited as useful by doctors when undertaking a fluid assessment include, blood pressure, heart rate and respiratory rate (Lloyd et al. 2019). Whereas increased heart rate is associated with prediction of AKI in patients in critical care (Wiersema et al. 2020), there is limited evidence to support the inclusion of respiratory rate when undertaking a fluid assessment in a patient with pre‐renal AKI. In a study by Macedo et al. (2010) involving 253 critically ill patients in 5 different centres, it was found that the severity of AKI could be underestimated due to dilution of serum creatinine and volume expansion. This is especially evident in patients with a background of chronic kidney disease. This means that complications, including elevated potassium, acidosis and fluid overload (which are associated with increased risk of death) (Libório et al. 2015) may not be identified until after prolonged renal damage. Hypotension has been cited as a statistically significant cause of AKI (Mehta et al. 2016) and, therefore, it would be expected that blood pressure in patients with AKI would be low. However, early warning scores (Potter et al. 2017) do not support this finding; this may be because there is a time delay between the episode of hypotension or dehydration and the development of AKI.

Whilst NEWS remains an important tool in the assessment and escalation of patients who are experiencing clinical deterioration, it has been found that it is not useful in predicting AKI or its severity (Riding 2019; Faisal et al. 2018; Potter et al. 2017). This means that clinical staff should be educated to understand that a patient who may have complications of AKI, such as fluid depletion, fluid overload, hyperkalaemia or acidosis, should not use the NEWS (or the updated version NEWS2) score as a basis for determining deterioration severity.

Wiersema et al. (2020) examined how predictive a clinical examination was in detecting AKI in patients in critical care and found that CRT on the sternum of > 4.5 s (Lima et al. 2009; Wiersema et al. 2020) was the most predictive, and is longer than the > 2 s recommended by Champion et al. (1981) as part of his trauma score (although this has long been debated). Subjectively cold peripheries were also found to be associated with prediction of AKI (Wiersema et al. 2020), and can be used to determine organ dysfunction severity (Lima et al. 2009). Although there are inherent risks with subjective assessment, peripheral temperature measurement may be a simple and useful strategy that could be undertaken. In a study involving patients with severe sepsis and septic shock in critical care, it was determined that the relationship between the toe to room temperature gradient statistically significantly correlated with other markers for tissue perfusion such as CRT on the knee and urine output (Bourcier et al. 2016)—which, as discussed previously, is an important indicator of kidney function. Other subjective assessments, that have been identified in dehydrated patients who had low blood pressure (in the supine position), include: dry mouth, increased thirst, dizziness and dry skin (Panuccio et al. 2020).

### 3.2 Implications for Practice

The findings from the scoping review (Table 3), indicate what should be included within a fluid assessment of a patient with AKI. Using a structured approach to fluid assessment will improve the management of the patient, which could limit the progression of AKI, length of stay and enhance outcomes.

**Table 3:** A fluid assessment in a patient with AKI should include the following elements.

Assessment	History taking
	Diagnosis
	Blood tests
	Urine output measurement
	Fluid balance charting
	Daily weights
Signs	Orthostatic hypotension: a drop in systolic BP of > 10 mmHg when standing
	Hypotension in the supine position of < 110 mmHg
	MAP ≤ 73 mmHg
	Raised heart rate
	CRT on the sternum > 4.5 s.
Symptoms	Subjectively cold peripheries
	Dry mouth,
	Increased thirst
	Dry skin.

This scoping review is informing a research study exploring assessment of fluid as well as the signs and symptoms of fluid overload and dehydration in patients with AKI.

## 4 Conclusion

This scoping review has presented a unique and important overview of the signs and symptoms that may be present in a patient with AKI and the clinical assessments that should be undertaken.

A rigorous fluid assessment in patients with AKI should be holistic and include history taking and diagnosis (NICE 2013), and this review points to the value of reviewing electrolytes, undertaking clinical assessments (and specifically CRT and blood pressure) and reviewing clinical signs and symptoms (reduced output and fluid overload). Urine output measurement and fluid balance charting remain an important element, with the use of daily weights as a crucial adjunct. Orthostatic hypotension, hypotension in the supine position and MAP ≤ 73 mmHg may indicate hypovolaemia; it is known that hypotension can lead to a reduction in kidney function and that this usually pre‐empts AKI. Other key clinical signs include elevated heart rate, prolonged CRT on the sternum (> 4.5 s) and subjectively cold peripheries. Clinical symptoms include dry mouth, increased thirst and dry skin.

Clinicians can use these findings to inform the management of patients with AKI—this has the potential to limit disease progression and enhance outcomes.

## Author Contributions

K.N. conceived, designed, conducted and wrote the review. N.P., L.W. and K.F. contributed to the design, and write‐up of the review. J.M. independently reviewed the papers selected for inclusion. All authors have reviewed the article.

## Conflicts of Interest

The authors declare no conflicts of interest.

## Supporting information


**PRISMA 2020 Flow Diagram: For Enhanced Fluid assessment study**.
