# Supplementary material for: Clinical Assessment of Fluid Status in Adults With Acute Kidney Injury: A Scoping Review
Source: J Ren Care. 2025 Apr 5;51(2):e70014. doi: 10.1111/jorc.70014 (PMC11971954; doi:10.1111/jorc.70014)
Supplement: Supplementary file 1 — PRISMA 2020 Flow Diagram: For Enhanced Fluid assessment study. [file JORC-51-0-s001.docx]

**Flow diagram 1**

**PRISMA 2020 Flow Diagram: For Enhanced Fluid assessment study**

Records removed before screening:

Duplicate records: (n=103)

Records not meeting inclusion criteria (pregnancy, children, animals) (n=155)

Records identified from PubMed (n=360), SCOPUS (n= 115), CINAHL (n=153): Databases (n=628). Searches ran 18.9.22 and reran 13.4.2024. Google scholar (n=12). Articles retrieved from reference list (n=15). Total articles (n=645)

## Identification

Records excluded
(n = 329)

Records screened
(n = 387)

Full text article sort for retrieval
(n =58)

## Screening

Articles assessed for eligibility
(n =58)

Studies excluded: 43

Reason 1: Not a primary study (n = 2)

Reason 2: Not focussing on concepts (n=41)

## Included

Studies included in review
(n =15)

*From:*  Page MJ, McKenzie JE, Bossuyt PM, Boutron I, Hoffmann TC, Mulrow CD, et al. The PRISMA 2020 statement: an updated guideline for reporting systematic reviews. BMJ 2021;372:n71. doi: 10.1136/bmj.n71

For more information, visit: <http://www.prisma-statement.org/>
